# Unbalanced YAP–SOX9 circuit drives stemness and malignant progression in esophageal squamous cell carcinoma

**DOI:** 10.1038/s41388-018-0476-9

**Published:** 2018-11-06

**Authors:** Lianghai Wang, Zhiyu Zhang, Xiaodan Yu, Xuan Huang, Zheng Liu, Yuhang Chai, Lei Yang, Qian Wang, Man Li, Jin Zhao, Jun Hou, Feng Li

**Affiliations:** 1 Department of Pathology, Immunology, and Key Laboratory of Xinjiang Endemic and Ethnic Diseases, Shihezi University School of Medicine, Shihezi, Xinjiang China; 2 Department of Pathology and Medical Research Center, Beijing Chaoyang Hospital, Capital Medical University, Beijing, China; 3 Department of Biomedical Sciences, Cornell University, Ithaca, NY USA; 4 Department of Stomatology, First Affiliated Hospital, Shihezi University School of Medicine, Shihezi, China

**Keywords:** Oncogenes, Prognostic markers, Oesophageal cancer

## Abstract

Yes-associated protein (YAP) has been identified as a key regulator of tissue homeostasis. However, the precise role and regulatory mechanism of YAP in esophageal squamous cell carcinoma (ESCC) remains unclear. Here we report that the genetic or pharmacological inhibition of YAP repressed cancer stem cell (CSC)-like properties, including tumorsphere-forming potential, cell motility, and chemoresistance in vitro, and was sufficient to attenuate tumor growth and CSC marker expression in ESCC xenografts. Mechanistically, YAP transcriptionally activated its downstream target SOX9 via TEAD1-mediated binding. We also observed a positive correlation between YAP signaling and SOX9 expression in two independent clinical cohorts. Intriguingly, YAP-targeting microRNAs, including miR-506-3p, which were induced by SOX9, post-transcriptionally repressed YAP expression, contributing to a negative feedback mechanism. Dual inhibition of YAP and SOX9 robustly suppressed malignant phenotypes. Notably, ESCC samples from The Cancer Genome Atlas (TCGA) dataset had frequent (44%) instances of YAP gene amplification and genetic inactivation of Hippo pathway regulators. Nuclear YAP expression was elevated in 197 ESCC tissues from a Chinese cohort. Together, our findings provide evidence that genetic hyperactivation of YAP unbalances the YAP–SOX9 feedback loop and confers CSC-like features in ESCC, suggesting that this YAP–SOX9 circuit represents a potential therapeutic target.

## Introduction

Esophageal cancer is the eighth most frequently diagnosed malignant tumor and the sixth leading cause of cancer-related deaths worldwide [1]. Squamous cell carcinoma comprises 80% of all cases of esophageal cancer and is more common in the developing world, especially in China, where cases account for approximately 70% of the global occurrence [2]. The prognosis of esophageal squamous cell carcinoma (ESCC) is poor, with a 5-year postoperative survival rate of 26.2% [3]. Therefore, it is critically urgent to identify effective diagnostic markers and novel therapeutic strategies.

The Hippo signaling pathway is a key regulator of organ size, tissue homeostasis, and stem cell self-renewal [4–6]. Transcriptional coactivators Yes-associated protein (YAP) and its paralog, transcriptional coactivator with PDZ-binding motif (TAZ; encoded by *WWTR1*), are the major downstream effectors of Hippo signaling [7] and are tightly regulated by upstream kinases such as mammalian STE20-like protein kinase 1 (MST1), MST2, large tumor suppressor 1 (LATS1), and LATS2, which phosphorylate and inactivate YAP/TAZ by causing their cytoplasmic retention or degradation. When Hippo signaling is silenced, hypophosphorylated YAP/TAZ translocate into the nucleus and induce target gene expression via interactions with cognate transcription factors, of which the most prominently studied are TEA domain family members 1–4 (TEAD1–4) [8]. Elevated YAP expression and nuclear accumulation are widespread in human cancers and have been shown to be essential for tumor initiation, progression, or metastasis [9–11]. Moreover, YAP/TAZ are active in cancer stem cells (CSC) and are functionally required for CSC expansion [12, 13]. Recently, focal amplifications of YAP gene locus at 11q22 have been identified in ESCC [14, 15]. However, the precise mechanisms for the expression and function of YAP in ESCC have not been elucidated.

In the present study, we found that YAP was overexpressed in ESCC tissues and was critical in regulating stemness. Importantly, we showed that frequent genetic hyperactivation of YAP disrupted the homeostasis of bidirectional regulation between YAP and SOX9, promoting stemness and ESCC progression. The YAP–SOX9 circuit could be targeted genetically or pharmacologically to inhibit the malignant progression of ESCC.

## Results

### YAP promotes CSC-like properties in esophageal cancer cells

**Fig. 1:**
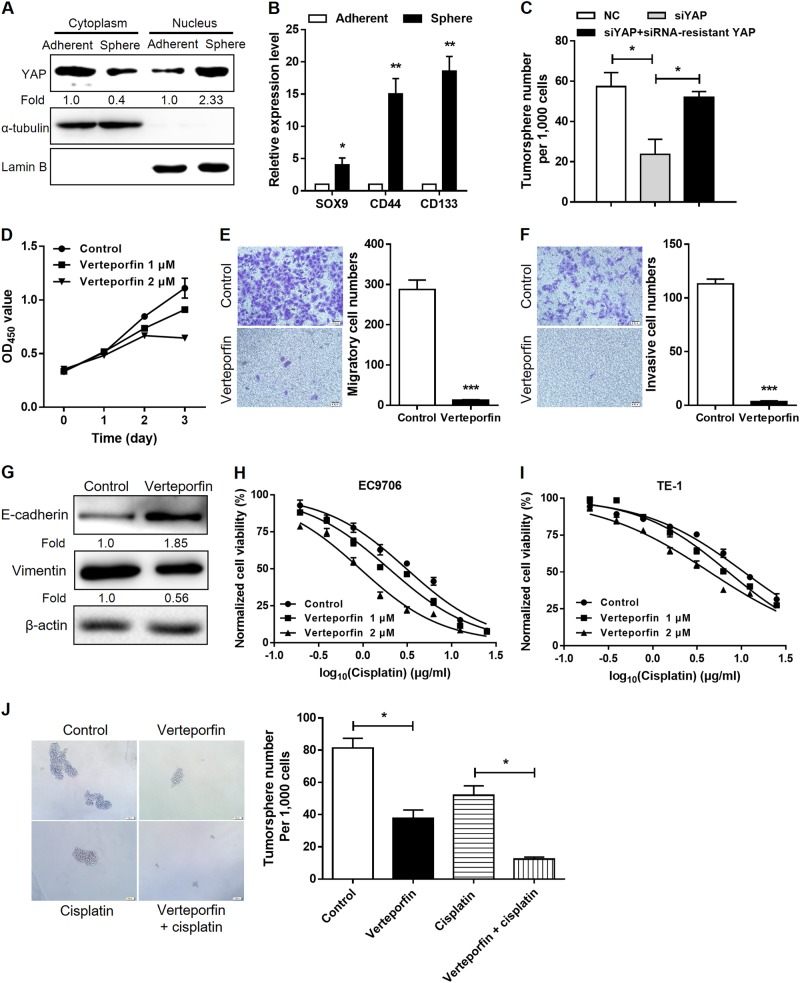
YAP promotes the acquisition of stem cell-like properties in ESCC cells. **a** Immunoblotting of nuclear and cytoplasmic YAP expression in Eca109 cells on two-dimensional adherent cultures or three-dimensional tumorspheres. α-Tubulin and Lamin B were used as loading controls. **b** Real-time PCR analysis of the indicated stemness-associated genes in Eca109 cells on adherent monolayers or tumorspheres. **c** Quantification of sphere numbers formed by Eca109 cells (per 1000 cells) after YAP knockdown and rescue by an siRNA-resistant mutant. **d** Growth curves for EC9706 cells measured by CCK8 assay after pharmacologic inhibition of YAP by verteporfin or treatment with vehicle control. **e**, **f** Effect of verteporfin (1 μM) on cell migration (**e**) and invasion (**f**) of EC9706 cells assessed using Transwell assay. **g** Immunoblotting of E-cadherin and vimentin expression in EC9706 cells after verteporfin (2 μM) treatment. β-Actin was used as a loading control. **h**, **i** Dose–response curves of EC9706 (**h**) and TE-1 (**i**) to cisplatin after verteporfin treatment. **j** Representative images (left) and quantification of tumorspheres (right) formed by Eca109 cells in the presence or absence of cisplatin (3 μg/ml) and verteporfin (1 μM). *P < 0.05, **P < 0.01

We observed that expression of nuclear YAP in Eca109 cells, together with the stemness-associated genes SOX9, CD44, and CD133, markedly increased when cells were cultured in three-dimensional suspension as tumorspheres compared with that for two-dimensional adherent monolayers (Fig. 1a, b). Next, we evaluated the effect of YAP loss-of-function through RNA interference. Three independent small interfering RNAs targeting YAP (siYAP) decreased its protein expression as expected (Fig. 3d). siYAP#2 resulted in the most robust reduction of YAP expression in EC9706 and TE-1 cells and was therefore chosen for subsequent assays. In nonadherent sphere formation assays, knockdown of YAP decreased tumorsphere numbers and size compared with that of the scramble control, whereas ectopic expression of an siRNA-resistant YAP (with synonymous mutations at the siRNA target site) rescued the tumorsphere-forming capacity (Fig. 1c). Verteporfin, which is an antagonist of YAP–TEAD association [16], significantly reduced cell proliferation (Fig. 1d), migration (Fig. 1e), and invasion (Fig. 1f). Enhanced cell motility and consequent invasion and metastasis are associated with epithelial-to-mesenchymal transition (EMT), which also confers CSC-like properties in tumor cells [17]. In accordance with reduced cell mobility, EMT was also inhibited in verteporfin-treated cells, as demonstrated by the increased expression of epithelial cell marker E-cadherin and decreased expression of mesenchymal marker vimentin (Fig. 1g). Resistance to chemotherapeutic drugs is one of CSC characteristics endowed to cancer cells [18]. We found that verteporfin treatment attenuated chemoresistance to cisplatin, a conventional chemotherapeutic drug for esophageal cancer, in EC9706 (Fig. 1h) and TE-1 cells (Fig. 1i). Consistent with YAP knockdown, verteporfin-treated Eca109 cells exhibited significantly decreased tumorsphere-forming capacity compared with that for cells treated with the vehicle control. Moreover, verteporfin synergistically reduced tumorsphere numbers and size with cisplatin treatment (Fig. 1j). These observations suggest that YAP is associated with the acquisition of CSC-like characteristics in ESCC.

### Pharmacologic inhibition of YAP attenuates tumor progression in vivo

**Fig. 2:**
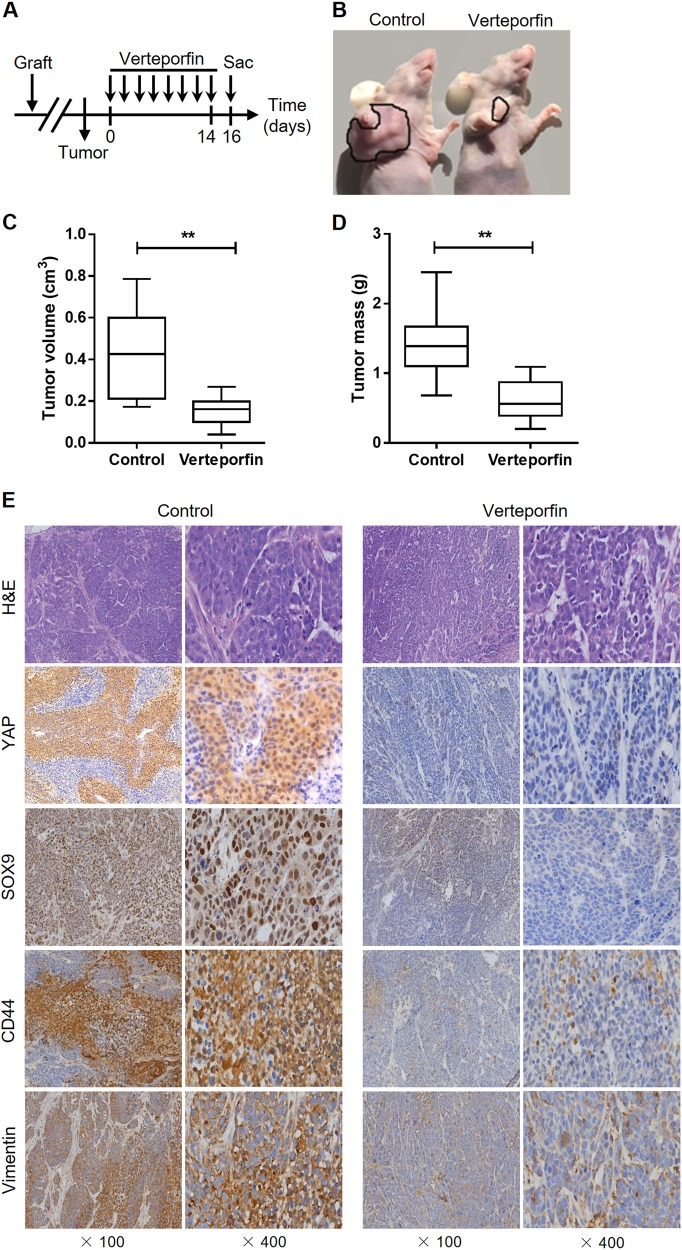
Treatment with verteporfin suppresses tumor growth in vivo. **a** Schematic diagram showing the experimental strategy for verteporfin therapy (*n* = 7 per group). **b**–**d** Tumors were excised from mice at the end of the experiment. Tumor volume (**c**) and weight (**d**) were measured. **e** Representative H&E and IHC staining of YAP, SOX9, CD44, and vimentin in ESCC xenografts after verteporfin treatment. **P < 0.01

To examine the biological significance of YAP in supporting tumor growth, we performed therapeutic intervention studies using verteporfin in ESCC xenograft model. Mice bearing EC9706-derived xenografts were intraperitoneally treated with verteporfin every 2 days for 2 weeks and sacrificed 2 days later (Fig. 2a). This treatment potently suppressed tumor growth, as measured by tumor volume and tumor weight, compared with the control treatment (Fig. 2b–d). Furthermore, immunohistochemical (IHC) staining of the tumor xenografts demonstrated that the levels of CSC markers SOX9 and CD44, as well as the mesenchymal marker vimentin, were diminished by the treatment (Fig. 2e), indicating that inhibition of YAP signaling decreases CSC-like properties in ESCC and therefore attenuates tumor growth.

### YAP activates SOX9 transcription through the TEAD1-binding site

**Fig. 3:**
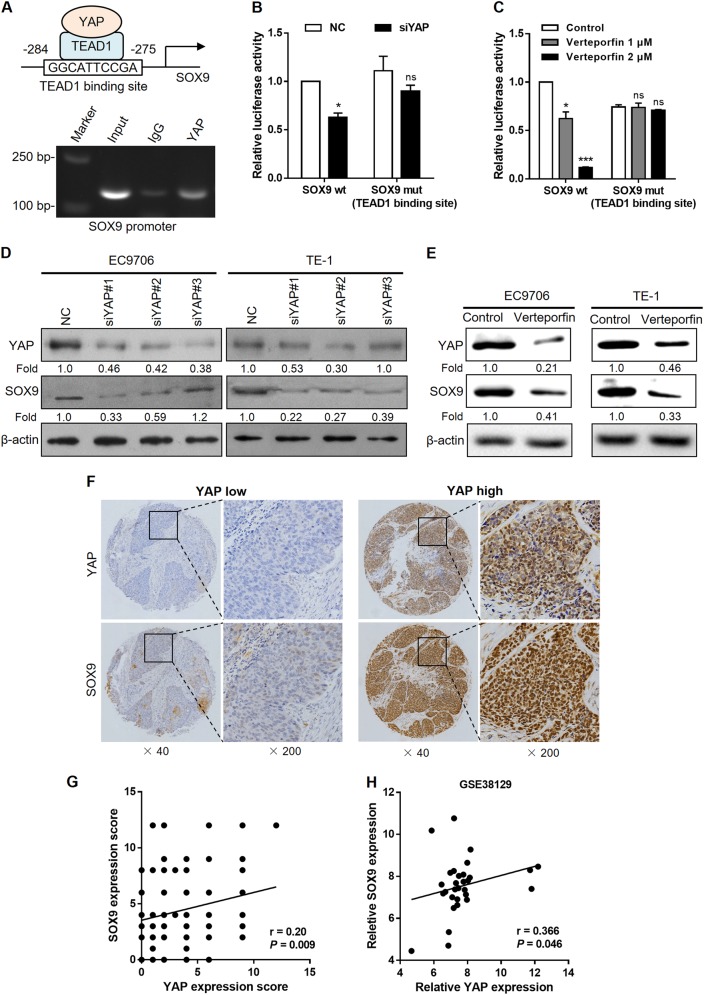
YAP activates SOX9 transcription through the TEAD1-binding site. **a** Predicted TEAD1-binding site with the highest prediction score in the proximal promoter region of SOX9 (top). ChIP assays with YAP antibody or control IgG were performed on chromatin from EC9706 cells using primers that amplify SOX9 promoter spanning the TEAD1-binding site (bottom). **b**, **c** Luciferase reporter assays in 293T cells transfected with SOX9 promoter reporter containing wild type (SOX9 wt) or mutated TEAD1-binding site (SOX9 mut) together with YAP siRNA (**b**) or verteporfin (**c**) treatment. **d**, **e** Immunoblot analysis of YAP and SOX9 expression in EC9706 cells (left) and TE-1 (right) after treatment with different siRNAs against YAP (**d**) or verteporfin at 2 μM (**e**). β-Actin was served as a loading control. **f** Representative images of SOX9 expression measured by IHC in YAP high and YAP low ESCC samples. **g** Correlation between YAP and SOX9 immunoreactivity scores in our sample set (*n* = 175), expressed using Spearman’s correlation coefficients. **h** Correlation between YAP and SOX9 expression in an independent ESCC dataset (*n* = 30). ns not significant, *P < 0.05, ***P < 0.001

Since YAP exerts its transcriptional coactivator function predominantly via interaction with transcription factor TEAD1 [19], we used the JASPAR database [20] to analyze the promoter region of several stemness-associated genes for TEAD1-binding sites. Notably, multiple TEAD1-binding sites were identified in the proximal promoter region of SOX9 (Supplementary Table 1). The binding site with the highest prediction score in the proximal promoter of SOX9 was chosen, and chromatin immunoprecipitation (ChIP) assay demonstrated that YAP was recruited to the SOX9 promoter at the TEAD1-binding site in EC9706 cells (Fig. 3a). The competency for TEAD1 binding was further examined using a luciferase reporter assay. YAP knockdown or verteporfin treatment reduced SOX9 promoter activity, whereas mutation of the TEAD1-binding site abrogated the effects of YAP depletion or verteporfin treatment (Fig. 3b, c). As expected, SOX9 protein expression was decreased in siYAP- or verteporfin-treated EC9706 and TE-1 cells (Fig. 3d, e). To determine whether YAP and SOX9 are coordinately expressed in clinical ESCC samples, we analyzed the expression patterns of SOX9 on the same tissue microarray. We found increased SOX9 expression by IHC in primary ESCC specimens with high levels of YAP (Fig. 3f). Quantification of this series of cases showed that SOX9 expression was positively associated with elevated nuclear YAP (*r* = 0.20, *P* = 0.009; Fig. 3g), which is consistent with the array data extracted from a publicly available ESCC dataset [21] (*r* = 0.366, *P* = 0.046; Fig. 3h).

### SOX9 modulates YAP expression through miR-506-3p

**Fig. 4:**
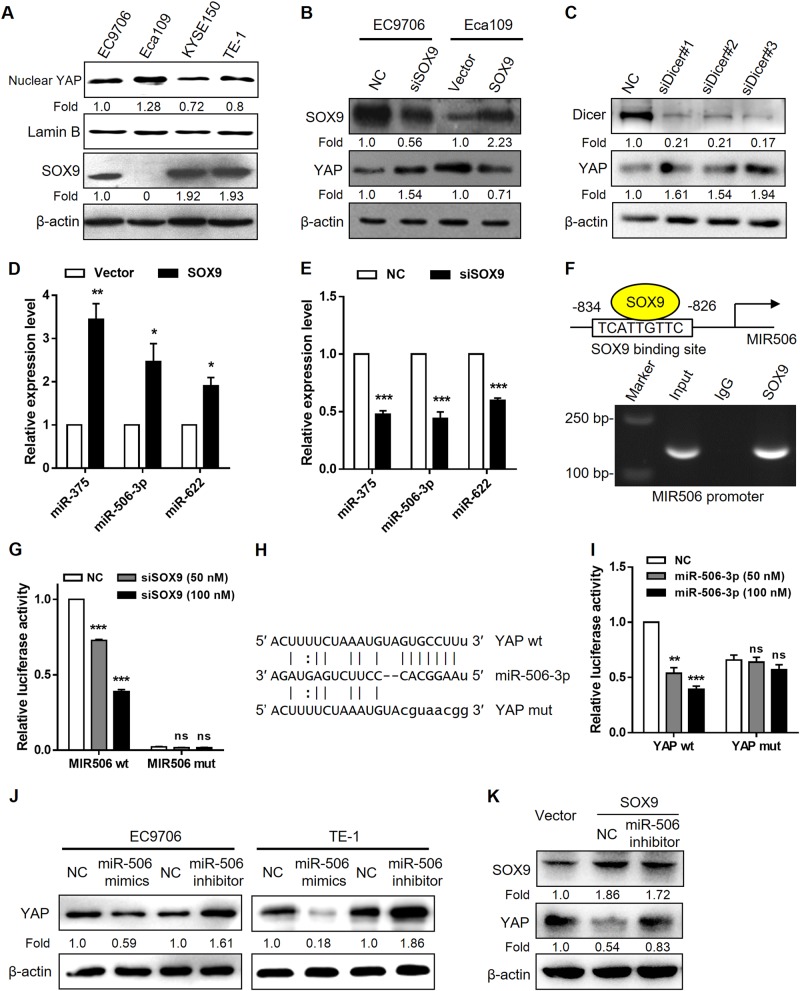
SOX9 post-transcriptionally regulates YAP expression through miR-506-3p. **a** Immunoblot analysis of endogenous SOX9 and nuclear YAP expression in a panel of ESCC cell lines. β-Actin and Lamin B were used as loading controls. **b** Western blot analysis for SOX9 and YAP protein levels after SOX9 knockdown in EC9706 cells and SOX9 overexpression in Eca109 cells. **c** Immunoblot analysis of Dicer and YAP expression in Eca109-SOX9 cells after treatment with different siRNAs against Dicer. **d**, **e** Real-time PCR analysis of the indicated miRNAs after SOX9 knockdown in EC9706 cells and SOX9 overexpression in Eca109 cells. miRNA levels were normalized to U6 expression. **f** Predicted SOX9-binding site with the highest prediction score in the proximal promoter region of MIR506 (top). ChIP assays with SOX9 antibody or control IgG were performed on chromatin derived from EC9706 cells. Primers flanking the SOX9-binding site in the *MIR506* 5′ region were used for PCR amplification (bottom). **g** Luciferase reporter assays in 293T cells transfected with MIR506 promoter reporter containing wild type (MIR506 wt) or mutated SOX9-binding site (MIR506 mut) together with siRNA against SOX9. **h** miR-506-3p target site was predicted within the 3′-UTR fragment of YAP using miRanda. Mutations in the seed region were highlighted in lowercase letters. **i** Luciferase reporter assays in 293T cells cotransfected with miR-506-3p mimics or scramble control and the indicated 3′-UTR reporters of YAP containing wild type (YAP wt) or mutated miR-506-3p target site (YAP mut). **j** Immunoblot analysis of YAP expression treated with miR-506-3p mimics or antagomirs in EC9706 (left) and TE-1 cells (right). β-Actin was served as a loading control. **k** Effect of antagonizing miR-506-3p concomitant with SOX9 overexpression on YAP protein levels in Eca109 cells. ns not significant. *P < 0.05, **P < 0.01, ***P < 0.001

Since a previous ChIP-seq study showed that there was a weak SOX9-binding peak associated with the YAP promoter in prostate cancer cells, indicating that SOX9 may activate YAP transcription [22], we next assessed whether SOX9 could regulate YAP expression in ESCC. Surprisingly, YAP protein levels increased after SOX9 knockdown in EC9706 cells and decreased after SOX9 overexpression in Eca109 cells (Fig. 4a, b), raising the question of whether SOX9 could post-transcriptionally control YAP expression. Intriguingly, YAP expression was rescued in Eca109-SOX9 cells after loss of miRNAs causing by Dicer knockdown (Fig. 4c). miRNA assay data indicated that several YAP-targeting miRNAs, including miR-375, miR-506-3p, and miR-622 [23–25], were upregulated in SOX9-overexpressing Eca109 cells compared with those of the vector control (data not shown). Quantitative real-time PCR analysis confirmed the significant increase in the levels of miR-375, miR-506-3p, and miR-622 in Eca109-SOX9 cells, as well as reduced miRNA levels after SOX9 knockdown (Fig. 4d, e). A putative SOX9-binding site with the highest score predicted by JASPAR was identified in the promoter region of *MIR506* (Supplementary Table 2 and Fig. 4f) and was pursued as a candidate for study in detail. Binding of endogenous SOX9 to the *MIR506* promoter was validated by ChIP assay (Fig. 4f). To confirm the transcriptional control of miR-506-3p by SOX9, promoter activity was investigated with the luciferase reporter assay. SOX9 knockdown suppressed the activity of *MIR506* promoter, whereas loss of the putative SOX9-binding site led to unresponsiveness to SOX9 repression (Fig. 4g). We then constructed luciferase reporter plasmids containing the YAP 3′-untranslated region (3′-UTR) fragment with wild type or mutated miR-506-3p-binding sites (Fig. 4h). Dual-luciferase reporter assays showed that miR-506-3p significantly suppressed the luciferase activities of the reporter containing the predicted miR-506-3p-targeting site but not the reporter with mutated targeting site (Fig. 4i), indicating that miR-506 directly targets *YAP* through the 3′-UTR region. In line with these data, overexpression of miR-506-3p repressed YAP protein expression, whereas treatment with the miR-506-3p inhibitor increased YAP protein levels in EC9706 and TE-1 cells (Fig. 4j). To further determine whether miR-506-3p acts as a SOX9 downstream effector on YAP protein expression, we performed a rescue experiment by antagonizing endogenous miR-506-3p in SOX9-overexpressing cells. Loss of miR-506-3p was accompanied by an increased YAP protein level (Fig. 4k). Collectively, these data suggest that SOX9 may play a role in the negative feedback regulation of YAP.

### Dual inhibition of YAP and SOX9 inhibits cell proliferation, EMT, and chemoresistance

**Fig. 5:**
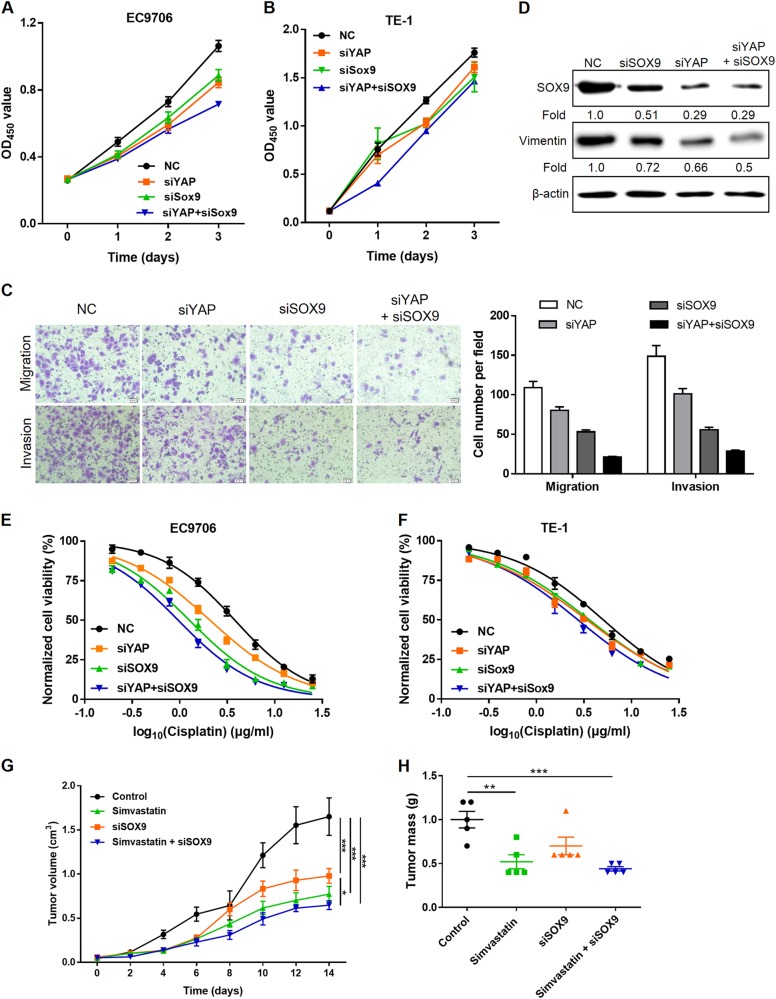
Silencing of YAP and SOX9 inhibits cell proliferation, EMT, and chemoresistance. **a**, **b** EC9706 (**a**) and TE-1 cells (**b**) were transfected with siYAP and siRNA against SOX9, and cell proliferation was determined using the CCK8 assay. **c** Effect of simultaneous knockdown of YAP and SOX9 on cell migration (up) and invasion (bottom) of EC9706 cells assessed using Transwell assay. **d** Immunoblotting of SOX9 and vimentin expression in EC9706 cells transfected with siYAP and siRNA against SOX9. β-Actin was used as a loading control. **e**, **f** Dose–response curves of EC9706 (**e**) and TE-1 cells (**f**) to cisplatin after simultaneous knockdown of YAP and SOX9. **g**, **h** Combination therapy of simvastatin and siSOX9 in established ESCC xenografts (*n* = 5 per group). Tumor volume was measured every other day (**g**), and tumor weights were measured at the end of the experiment (**h**). *P < 0.05, **P < 0.01, ***P < 0.001 by two-way (**g**) or one-way (**h**) ANOVA, Tukey post hoc test

We further examined the impacts of dual inhibition of YAP and SOX9 on cells growth, motility, and chemoresistance to evaluate whether inhibition of SOX9 could potentiate the antitumor effect of YAP repression. As expected, siYAP in combination with SOX9 depletion robustly attenuated cell proliferation in EC9706 and TE-1 cells compared with that of siYAP treatment alone (Fig. 5a, b). Similar effects of dual inhibition on cell motility, as measured by cell migration and invasion (Fig. 5c) and EMT markers (Fig. 5d), were also observed. Simultaneous knockdown of SOX9 and YAP further sensitized EC9706 and TE-1 cells to cisplatin treatment (Fig. 5e, f). Moreover, the combination of simvastatin, which restricts YAP nuclear accumulation [40], and cholesterol-conjugated SOX9 siRNA treatment exhibited a strong inhibition of EC9706-derived xenograft growth in mice (Fig. 5g, h).

### YAP is genetically hyperactivated and overexpressed in ESCC

**Fig. 6:**
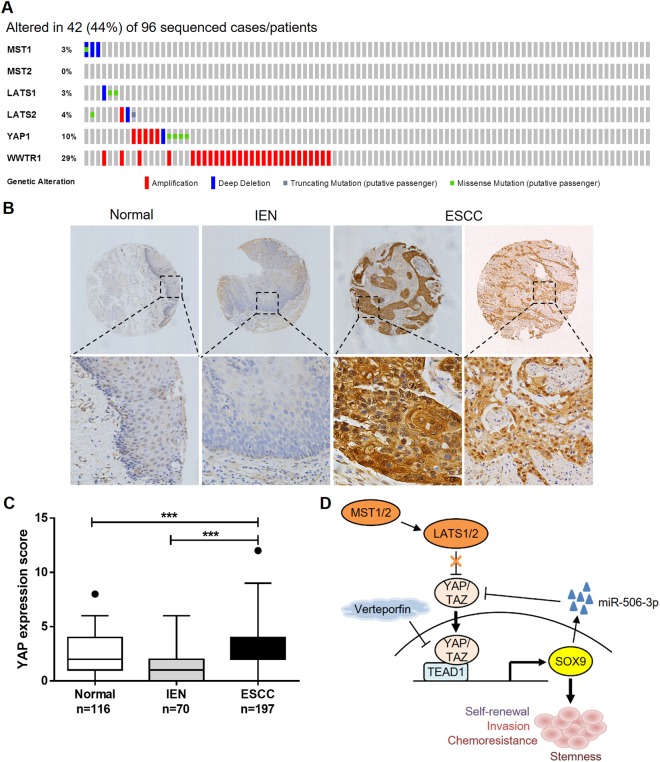
YAP is genetically hyperactivated and overexpressed in ESCC. **a** A visual summary of the alteration frequencies of major components involved in the Hippo/YAP pathway across the ESCC samples in the TCGA Esophageal Carcinoma dataset. Each row represents a gene, and each column corresponds to a sequenced case (*n* = 96). Note the frequent deletion and mutation of *MST1/2* and *LATS1/2* and amplification of *YAP* and *WWTR1*. **b** Representative staining of YAP expression in ESCC tissues, adjacent normal squamous epithelium, and intraepithelial neoplasia (IEN). **c** Statistical analysis of IHC-determined YAP expression in normal, IEN, and tumor tissues. ***P < 0.001 by Kruskal-Wallis with Dunn's post hoc test. **d** A proposed model for an unbalanced YAP–SOX9 regulatory circuit in promoting ESCC progression.

To investigate the clinical significance of elevated YAP expression/activation in ESCC, we analyzed genetic alterations in the Hippo/YAP pathway in the ESCC samples from The Cancer Genomic Atlas (TCGA) Esophageal Carcinoma dataset using the cBioPortal for Cancer Genomics [26]. In the 96 ESCC patients, 44% cases have alterations in at least one of the components in the Hippo/YAP pathway. *MST1/2* and *LATS1/2*, upstream regulators of the Hippo tumor-suppressing signaling, are frequently deleted and mutated, whereas the downstream effectors, *YAP* and *WWTR1* (encoding TAZ), are frequently amplified (Fig. 6a). To validate that YAP plays a role in ESCC progression, we examined the expression pattern of YAP using IHC on a human tissue microarray containing 197 cases of ESCC together with their nontumor counterparts. Nuclear staining of YAP was weak or absent in adjacent nontumor esophageal tissues, whereas YAP immunosignal was strong in the nucleus of tumor cells (Fig. 6b). Compared with normal squamous epithelium and intraepithelial neoplasia, the mean immunoreactivity scores were significantly higher in the ESCC tissues (Fig. 6c).

## Discussion

The findings presented here support the existence of a feedback loop involving YAP/SOX9/miR-506-3p in regulating the proliferation and motility of ESCC cells, which plays a critical role in cancer progression. In normal esophagus development and homeostasis, low levels of nuclear YAP transcriptional coactivate the pluripotency transcription factor SOX9 via interaction with TEAD1, which in turn initiates a negative feedback regulation at the post-transcriptional level through miR-506-3p to restrain YAP signaling in normal esophageal squamous epithelial cells. However, in ESCC tissues, frequent YAP gene amplification and genetic inactivation of Hippo pathway regulators led to aberrant hyperactivation of YAP and disruption of homeostasis, inducing stemness and subsequent cancer development (Fig. 6d).

The Hippo/YAP signaling pathway is involved in the regulation of ESCC initiation and progression, indicated by the frequent genomic amplification of the *YAP* locus and deletion/mutation of the core Hippo kinases *MST* and *LATS* obtained from the TCGA genomic datasets of ESCC patient samples (Fig. 6a). Frequent inactivating mutations were also identified in Hippo pathway regulators *FAT1*, *FAT2*, *FAT3*, and *FAT4* (27%) or *AJUBA* (7%) by exome sequencing on a large cohort of northern Chinese cases [14], providing a genetic mechanism for high-level nuclear YAP expression/activation in ESCC. However, since non-Hippo-dependent YAP regulation has also been reported [27, 28], the possibility that the pro-proliferative role of YAP in ESCC cells could be independent of the Hippo signaling warrants further study.

In this study, we found that YAP was almost absent from normal esophageal epithelium, whereas strong nuclear staining of YAP was observed in fully developed ESCC but not precancerous lesions. Until now, two IHC studies have shown that nuclear YAP may serve as a predictive marker for worse outcome in ESCC [29, 30]. YAP overexpression was also associated with poor prognosis and conferred CSC-like properties in hepatocellular carcinoma [31]. Unexpectedly, Kaplan-Meier analysis of the TCGA Esophageal Carcinoma dataset indicated that adenocarcinoma patients with high level of YAP mRNA had longer overall survival, although no significant association was observed in squamous cell carcinoma patients (Supplementary Figure 1). The potentially prognostic role of YAP mRNA expression and nuclear accumulation is worth further investigation.

SOX9, a high-mobility group box transcription factor, has been consistently identified as a CSC marker in a variety of cancers, where it enhances tumorigenesis through stimulation of proliferation and self-renewal [32–34]. Our current data and previous report in esophageal adenocarcinoma [13] suggest that activation of SOX9 by YAP via TEAD-mediated transcription might be the uniform driver for endowing CSC-like properties in both histological subtypes of esophageal carcinoma. ESCC cells with YAP knockdown exhibited decreased SOX9 expression, attenuated sphere-forming potential, and resistance to cytotoxic drugs. YAP-targeted therapy also diminished SOX9 expression and tumor growth in ESCC xenograft.

Cell-intrinsic negative feedback loops are important to ensure proper physiological regulation and homeostasis of the cells. Here, we showed that SOX9 stimulated the transcription of YAP-targeting miRNAs including miR-506-3p to inhibit YAP translation, constituting a negative feedback regulation of the YAP signaling. We found that SOX9 played two pivotal functional roles, as one of the downstream targets of YAP and as an indirect upstream suppressor of YAP. While our findings indicated that SOX9 negatively regulated YAP expression, SOX9 binding to YAP promoter has been reported to stimulate the transcription of YAP in prostate cancer cells [22], suggesting diverse, context-specific regulatory roles of SOX9 [35].

YAP/TAZ are fundamental for tumorigenesis but unimportant for adult tissue homeostasis, making them appealing therapeutic targets in tumors [6]. Since TEAD factors have been reported to be required for YAP/TAZ transcriptional responses in most cellular contexts, inhibiting the YAP/TAZ–TEAD interaction represents one of the most promising strategies for anti-YAP/TAZ therapies [6]. Verteporfin, a clinical photosensitizer for the treatment of macular degeneration, disrupts the interaction between YAP and TEAD, thus abrogating YAP-induced transcription [36]. In line with previous studies [37, 38], we showed that cancer cells were sensitized to a cytotoxic drug and tumor growth was pharmacologically blocked by verteporfin treatment in ESCC, demonstrating the therapeutic potential of inhibiting the YAP–TEAD interaction in tumors with aberrant YAP activation. Other inhibitors that suppress YAP function might also harbor therapeutic benefits in cancers [39]. Indeed, substantial evidence indicates that statins, a class of commonly used anti-atherosclerotic drugs, which inhibit the mevalonate cholesterol biosynthesis pathway, have anticancer effects through restricting YAP nuclear accumulation and thus activity in cancers [40, 41]. However, while simultaneous inhibition of YAP and SOX9 was more robust at silencing this YAP–SOX9 circuit as indicated by suppressed cell proliferation, EMT, and chemoresistance in vitro, new pharmacologic agents targeting SOX9 and efficient drug delivery are needed for combination treatment in vivo. Moreover, YAP-inactivating therapy might yield optimal outcomes when combined with traditional chemotherapies targeting proliferative tumor bulk.

In summary, we found that SOX9 is, at the same time, a downstream target and an upstream regulator of YAP signaling. The complex regulatory circuit between YAP and SOX9 provides feedback regulation of YAP and thus ensures tissue homeostasis. Frequent aberrant hyperactivation of YAP breaks this feedback loop and disrupts tissue homeostasis in esophageal cancer. Our study indicates that targeting of the YAP–SOX9 signaling circuit represents a novel therapeutic strategy for ESCC.

## Materials and methods

### Cell culture

ESCC cell lines (Eca109, EC9706, and TE-1) and 293T cells were obtained from the Institute of Biochemistry and Cell Biology of the Chinese Academy of Sciences (Shanghai, China) and maintained in RPMI 1640 medium supplemented with 10% fetal bovine serum. For tumorsphere formation, Eca109 cells were suspended in DMEM/F12 medium supplemented with 20 ng/mL EGF (Peprotech), 2% B27 (Gibco), and 20 ng/mL basic FGF (Peprotech) and seeded in ultra-low adhesion plates (Corning). Tumorspheres were visualized under a light microscope and counted in five random fields after 7-day cultivation.

### Reagents

YAP antibody (#14074) was from Cell Signaling Technology. SOX9 (#AB5535) antibody was from Millipore. Antibodies against E-cadherin (sc-8426) and vimentin (sc-6260) were purchased from Santa Cruz Biotechnology. Anti-CD44 (EP44) and anti-vimentin (OTI5D7) antibodies were from ZSGB-BIO. siRNA against human YAP and SOX9, mimics and antisense antagomirs of miR-506-3p, and scramble control RNA oligos were purchased from GenePharma.

### Real-time PCR

Total RNA was isolated using Total RNA Kit I (OMEGA) and reverse transcribed. Real-time PCR was performed on a 7500 Fast Real-Time PCR System (Applied Biosystems) using Fast SYBR qPCR mixture (CWBIO). Primer sequences are listed in Supplementary Table 3. GAPDH or U6 was used as a reference gene.

### Cell proliferation/viability

Cell proliferation/viability was measured using the cell counting kit-8 (CCK-8) as described previously [42]. Absorbance at 450 nm (OD_450_) was measured in a microplate reader (BIO-RAD xMark).

### Transwell assay

In vitro migration and invasion assays were performed using a Transwell chamber according to our previous work [42]. Migrated or invaded cells were fixed, stained with 1% crystal violet, and counted using a light microscope in three random fields.

### ChIP analysis

ChIP assays were carried out using the EZ-ChIP kit (Millipore) according to the manufacturer’s instructions, using the antibody against YAP or SOX9. The amount of immunoprecipitated DNA was analyzed by PCR using SOX9 promoter primers (spanning the TEAD1-binding site): forward: 5′-GCAGTGAAAAGAAATGTCGGAGG-3′; reverse: 5′-TTCCAAGTGTGTAAGTTTGTCGT-3′ or MIR506 promoter primers (spanning the SOX9-binding site): forward: 5′-GCATTGCCCTATTTTGTGAGCA-3′; reverse: 5′-GAAGGCCTACAGCAGCAGAA-3′.

### Luciferase reporter assay

293T cells were seeded in 24-well plates and transfected with the indicated RNA oligos together with the pGL3-SOX9 promoter reporter plasmid, pGL4.10-MIR506 promoter reporter, or pMIR-REPORT-YAP 3′-UTR plasmid and a Renilla luciferase vector for normalization. Relative luciferase activity was determined using the Dual-luciferase Reporter Assay System (Promega).

### Human ESCC samples

Formalin-fixed paraffin-embedded tissue blocks from 197 patients who underwent esophagectomy without prior chemotherapy or radiotherapy were collected between 2004 and 2013 at the First Affiliated Hospital of Shihezi University School of Medicine, Xinjiang Yili Prefecture Friendship Hospital, and the People’s Hospital of Xinjiang Uyghur Autonomous Region. Subjects were recruited with written informed consent and with approval from the Ethics Committee of the First Affiliated Hospital, Shihezi University School of Medicine.

### IHC analysis

IHC staining was performed as previously described [43]. Briefly, paraffin sections underwent antigen retrieval and were incubated with primary antibodies overnight at 4 °C, followed by appropriate secondary antibodies. Immunostaining degree of each sample was scored by pathologists who were blinded to the clinicopathologic data, based on nuclear staining intensity (0, negative; 1, weak; 2, moderate; 3, strong) and percentage of positive cells (0, <5% positive cancer cells; 1, 6–25% positive cancer cells; 2, 26–50% positive cancer cells; 3, 51–75% positive cancer cells; 4, ≥76% positive cancer cells). The final immunoreactivity score is the product of the intensity score and the extent score.

### Xenograft tumor-formation assay and therapeutic treatment

Female BALB/C nude mice (Beijing Vital River Laboratory Animal Technology) at 4–5 weeks of age were used in this research. EC9706 cells grown at logarithm phase were subcutaneously inoculated into the armpit of mice to establish ESCC xenografts. For therapeutic treatment, mice bearing established xenografts were randomly divided and delivered verteporfin (50 mg/kg body weight; intraperitoneally), simvastatin (50 mg/kg body weight; intraperitoneally), cholesterol-conjugated siSOX9 (5 nM; intratumorally), simvastatin plus cholesterol-conjugated siSOX9, or PBS as a control every other day for 2 weeks. Tumor volume was measured with a caliper and calculated using the formula: length × width^2^ × *π*/6 [33]. At the end of the experiments, the mice were sacrificed, and the tumors were excised, weighed, fixed, and paraffin-embedded. All procedures were performed without blinding and with approval from the Animal Experimental Ethical Inspection of First Affiliated Hospital, Shihezi University School of Medicine.

### Statistical analysis

No statistical methods were used to estimate sample size, and no samples or animals were excluded from the analysis. Numerical data are presented as the mean ± SEM. Comparisons between two groups were conducted using two-tailed Student’s *t*-test. Significance of the association between YAP and SOX9 was evaluated using nonparametric Spearman correlations. Differences between values were considered statistically significant when *P* < 0.05.

## Electronic supplementary material

Supplementary Tables

Supplementary Figure
