# Supplementary material for: Unbalanced YAP–SOX9 circuit drives stemness and malignant progression in esophageal squamous cell carcinoma
Source: Oncogene. 2018 Nov 6;38(12):2042–55. doi: 10.1038/s41388-018-0476-9 (PMC6756096; doi:10.1038/s41388-018-0476-9)
Supplement: Supplementary file 1 — Supplementary Tables [file 41388_2018_476_MOESM1_ESM.doc]

**Supplementary Table 1.** Predicted binding sites of TEAD1 in the SOX9 promoter.

| Score | Relative score | Start^a^ | End | Predicted site sequence |
| --- | --- | --- | --- | --- |
| 9.062 | 0.910 | -284 | -275 | GGCATTCCGA |
| 8.015 | 0.888 | -1675 | -1666 | AACATCCCAT |
| 7.239 | 0.872 | -670 | -661 | TGCTTTCCAT |
| 6.073 | 0.848 | -234 | -225 | CGCCTTCCCC |
| 6.051 | 0.848 | -131 | -122 | CACAATCCTC |

^a^ position relative to the transcription initiation site (defined as the +1 position) of SOX9, e.g., -284 means an element located 284 nucleotides upstream of SOX9 transcription start site.

**Supplementary Table 2.** Predicted binding sites of SOX9 in the MIR506 promoter.

| Score | Relative score | Start^a^ | End | Predicted site sequence |
| --- | --- | --- | --- | --- |
| 10.639 | 0.932 | -834 | -826 | TCATTGTTC |
| 9.679 | 0.904 | -1297 | -1289 | TTATTGTTT |
| 7.874 | 0.853 | -1103 | -1095 | ATATTGTTA |
| 7.517 | 0.843 | -1222 | -1214 | GCATTGTGT |
| 7.409 | 0.840 | -319 | -311 | CTATTGACT |

^a^ position relative to the transcription initiation site (defined as the +1 position) of MIR506, e.g., -834 means an element located 834 nucleotides upstream of MIR506 transcription start site.

**Supplementary Table 3. Oligonucleotide sequences for real-time PCR.**

| Primer name | Sequence |
| --- | --- |
| SOX9 forward | TCCTCAGGCTTTGCGATTT |
| SOX9 reverse | TGCTCGGGCACTTATTGG |
| CD44 forward | ACTGTTATATCAGAGGAGTAGGAGA |
| CD44 reverse | ACAGCACAAGAATGAACAATGG |
| CD133 forward | CATTGACTTCTTGGTGCTGTTGA |
| CD133 reverse | CTGCGTGAAGAATATGCTGTAGG |
| GAPDH forward | GCACCGTCAAGGCTGAGAAC |
| GAPDH reverse | TGGTGAAGACGCCAGTGGA |

Supplementary Figure 1. Kaplan-Meier analysis of YAP mRNA expression and overall survival of patients from the TCGA Esophageal Carcinoma dataset using the UCSC Xena browser (http://xena.ucsc.edu). (A) Total esophageal carcinoma. (B, C) Subgroup analyses by histological type.
