# Supplementary figures and images for: Unbalanced YAP–SOX9 circuit drives stemness and malignant progression in esophageal squamous cell carcinoma
Source: Oncogene. 2018 Nov 6;38(12):2042–55. doi: 10.1038/s41388-018-0476-9 (PMC6756096; doi:10.1038/s41388-018-0476-9)

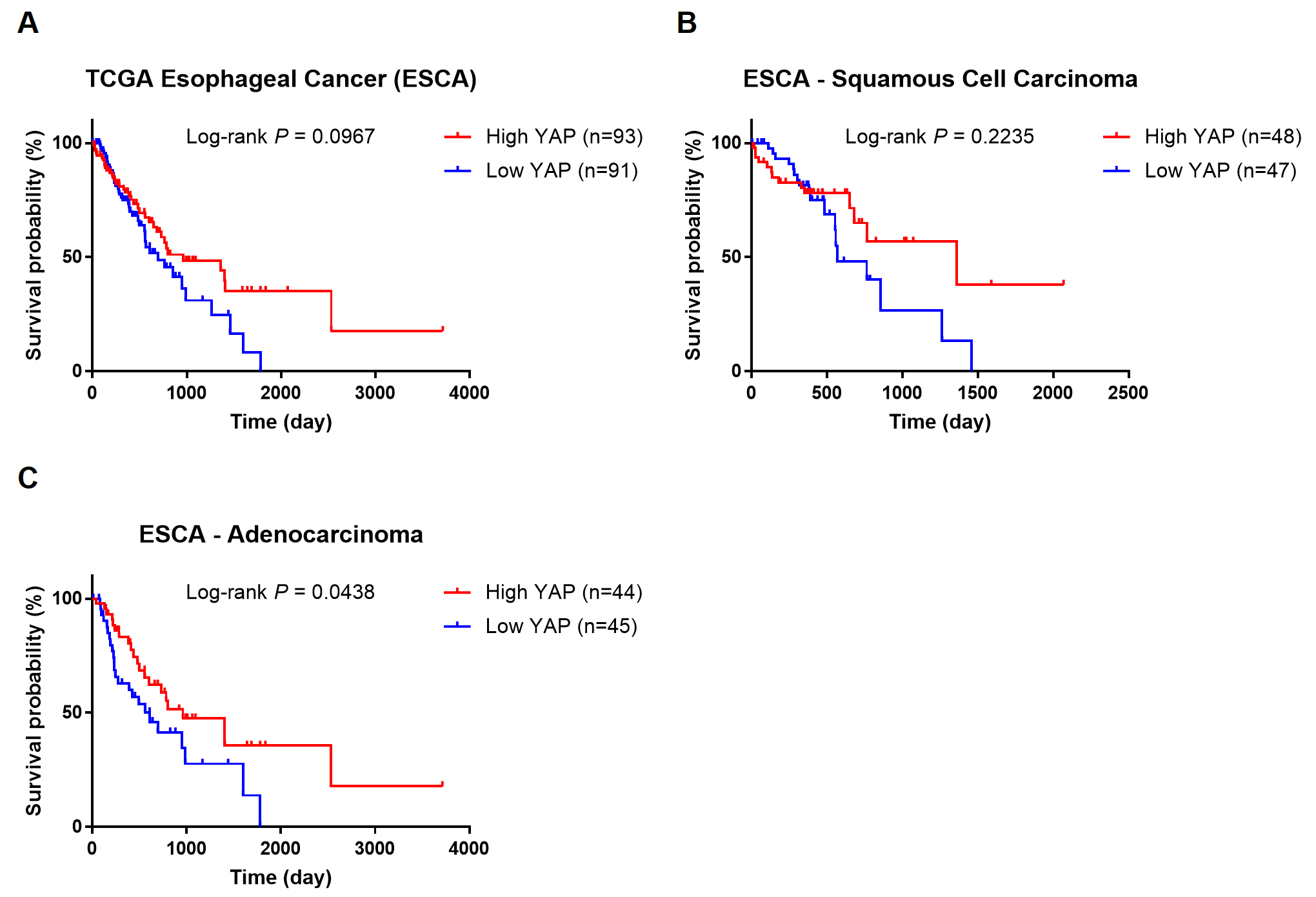

Supplement: Supplementary file 2 — Supplementary Figure [file 41388_2018_476_MOESM2_ESM.tif]
